# Management of Peri-Implantitis Lesions without the Use of Systemic Antibiotics: A Systematic Review

**DOI:** 10.3390/dj8030106

**Published:** 2020-09-14

**Authors:** Ahsen Khan, Ankit Goyal, Scott D. Currell, Dileep Sharma

**Affiliations:** 1 College of Medicine and Dentistry, James Cook University, 14-88 McGregor Road, Smithfield, QLD 4878, Australia; ahsen.khan@my.jcu.edu.au (A.K.); ankit.goyal@my.jcu.edu.au (A.G.); scott.currell@my.jcu.edu.au (S.D.C.); 2 Australian Institute of Tropical Health and Medicine, James Cook University, Cairns, QLD 4878, Australia

**Keywords:** peri-implantitis, decontamination, debridement, nonsurgical therapy

## Abstract

Background: This systematic review aims to assess the current evidence on the efficacy of surgical and non-surgical debridement techniques in the treatment of peri-implantitis lesions without the use of any antimicrobials. Method: Five electronic databases (MEDLINE, Pubmed, Scopus, CINAHL and Cochrane) were used, alongside hand searches, to find relevant articles. Full-text articles that were randomised controlled trials, published in the English language from 2011 onwards without pre-operative, peri-operative and post-operative antibiotic usage were included. The study was conducted according to the latest Preferred Reporting Items for Systematic Reviews and Meta-Analyses (PRISMA)-P protocols, the latest Cochrane Risk of Bias tool and each investigated intervention was evaluated using the grading of recommendation, assessment, development and evaluation (GRADE) system. Results: The search yielded 2718 results. After initial screening, 38 full-text articles were assessed for eligibility. From these, 11 studies satisfied all inclusion criteria. These 11 articles described six non-surgical and five surgical debridement therapies. Most articles were classified as having either a high risk of bias or presenting with some concerns. Small sample sizes, in combination with this risk of bias, meant that all interventions were adjudged to be of either low or very low quality of evidence. Conclusion: While all investigated modalities displayed some sort of efficacy, this review suggests that a surgical approach may be best suited to treating peri-implantitis lesions in the absence of antibiotic therapy. Despite this weak indication, further research is required in this field.

## 1. Introduction

Over the last four decades, dental implants (DI) have revolutionised the treatment of edentulous and partially dentate patients alike. Current practice of DI placement demonstrates high success rates [1]. Despite favourable success rates, DI are still subject to failures due to a variety of reasons. One of the leading causes of failure is biological complications. Biological complications occur when bacterial plaque accumulates around an implant, which consequently causes inflammatory changes in the tissues surrounding the implant. When this inflammatory process is limited to the soft tissues, the condition is known as peri-implant mucositis and when it spreads to the underlying alveolar bone, it is known as peri-implantitis [2,3,4].

In 2017, the American Academy of Periodontology and the European Federation of Periodontology collaborated to present an update on peri-implant diseases and conditions [5,6,7]. The workshop defined peri-implantitis as “a plaque-associated pathological condition occurring in tissues around DI, characterized by inflammation in the peri-implant mucosa and subsequent progressive loss of supporting bone” [5].

Clinical features of peri-implantitis lesions include the presence of bleeding on probing (BoP) and/or suppuration on probing (SoP); increased peri-implant probing depths (PPD) and/or mucosal recession (MR) and radiographic marginal bone loss (MBL) compared to previous examinations [5]. The prevalence of peri-implant pathology has been shown to be positively correlated to the duration for which the implant has been in function [8]. With an increasing number of DI being placed annually, the prevalence of peri-implantitis is expected to increase proportionally. Peri-implantitis is expected to affect 63.4% of all patients and 30.7% of all functional DI [9]. While there have been several clinical studies demonstrating clinical resolution of peri-implantitis lesions, a ‘gold standard’ protocol for treatment is yet to be established [1,10]. Management of peri-implantitis lesions are based on non-surgical and surgical approaches (Table 1).

Over the last decade, there have been several reviews investigating the efficacy of non-surgical therapies [12], surgical therapies [13,14,15], or both [1,16,17,18]. Furthermore, multiple reviews have investigated the efficacy of adjunctive treatment modalities, such as the use of dental laser [19], air-abrasive system [20], or antibiotics [21].

However, to the best of the authors’ knowledge, there no published reviews on the efficacy of debridement and decontamination techniques, not involving concurrent antibiotic therapy. As the accumulation of bacterial plaque plays a critical role in the pathogenesis of peri-implantitis, the need for implant debridement and/or decontamination to eliminate pathogenic bacterial flora becomes obvious [11]. The use of antibiotics as an adjunctive treatment modality can have a positive effect on treatment outcomes, however, it confounds the effectiveness of any given therapy. Of late, the usage of antibiotics has become controversial due to a global concern around increasing antibiotic resistance [22]; it is important to establish the most effective surgical and non-surgical debridement and/or decontamination technique, thereby eliminating the need for exogenous antibiotic agents. Thus, this systematic review aims to assess the efficacy of debridement and decontamination protocol, with or without adjunctive treatment, in the absence of any exogenous antibiotic treatment in the treatment of peri-implantitis lesions through the use of either surgical or non-surgical techniques.

## 2. Materials and Methods

### 2.1. Protocol and Registration

This systematic review was prepared in accordance with the Preferred Reporting Items for Systematic Review and Meta-Analyses Protocols (PRISMA-P) [23] and the Cochrane Handbook for Systematic Reviews of Interventions [24]. This review protocol has been registered with PROSPERO (Record: CRD42019116378), as recommended by Booth et al. [25,26].

### 2.2. Eligibility Criteria

The inclusion and exclusion criteria were based on the population, intervention, comparator, outcome and study design (Table 2).

### 2.3. Information Sources, Search Strategy and Study Selection

Five electronic databases, MEDLINE, PubMed, Cochrane Library, CINAHL and Scopus, were searched using Medical Subject Headings (MeSH) terms, key words and Boolean operators. Searches did not have any limitations placed on them to enable the widest pool of studies to be captured. The search strategy for Medline is presented in Table 3. The full electronic search strategy is accessible on PROSPERO (https://www.crd.york.ac.uk/prospero/display_record.php?ID=CRD42019116378).

**Figure 1:**
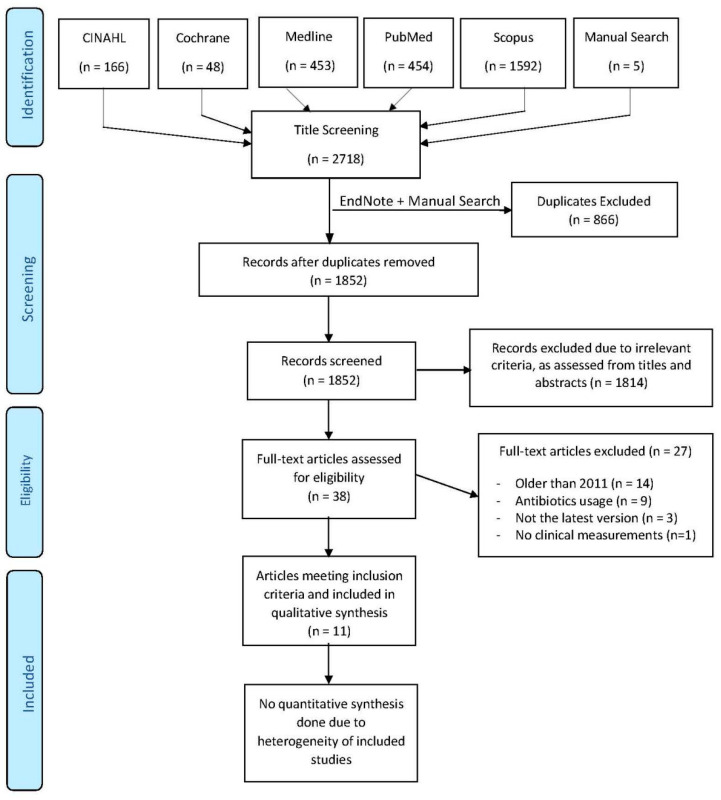
Prisma Flow chart of the review process.

Eight journals (Journal of Clinical Periodontology, Journal of Periodontology, International Journal of Implant Dentistry, Clinical Implant Dentistry and Related Research, Clinical Oral Investigations, Clinical Oral Implants Research and Implant Dentistry) were selected to conduct manual searches to ensure that additional articles were not missed in the electronic search. The World Health Organization’s International Clinical Trials Registry Platform and Clinical trials database (Clinicaltrials.gov) were scanned to identify any relevant ongoing trials. Grey literature databases such as Opengrey and System for Information on Grey Literature in Europe was also searched for any relevant grey literature. Reference lists of review articles and all included studies were checked for any further articles of relevance. A reference management software (Endnote version X8; Clarivate Analytics, Philadelphia, PA, USA) was used to sort the results and remove duplicates. Searches were initiated in June 2019 (Figure 1).

Relevant full-text articles were identified through their titles and abstracts, before being further assessed according to the pre-specified inclusion and exclusion criteria. Data extraction of included studies was then conducted and collated into a table format.

### 2.4. Risk of Bias (RoB) in Individual Studies

The 2019 Cochrane Collaboration bias assessment tool was used to assess the RoB of included studies [24]. This tool allowed analysis of each study across five key domains to adjudge each study as having either low, concerning or high RoB.

### 2.5. Data Synthesis: Evaluating the Effect of the Intervention

The primary end-point of interest with these studies was to find the intervention which causes the greatest resolution of peri-implantitis lesions. To assess this, the association/difference between different modalities and successful treatment outcomes were compared. The Grading of Recommendations, Assessment, Development and Evaluations (GRADE) approach was used to assess the quality of evidence for each treatment modality [27]. The parameters used in the GRADE approach (RoB, inconsistency, indirectness, imprecision or publication bias) were downgraded by one level wherever serious concern was found. An overall quality of evidence (high, moderate, low or very low quality) could then be adjudged.

### 2.6. Additional Analysis

Cohen’s kappa statistic was used to determine the inter-rater agreement between review authors during article selection and RoB and GRADE assessments.

## 3. Results

### 3.1. Literature Search and Screening Process

Using the search strategy described above, 2718 articles were found. After removing duplicates and the addition of five additional hand-searched articles, title and abstract evaluation resulted in 38 full-text articles meeting pre-specified inclusion and exclusion criteria. Following further evaluation, it was determined that 11 articles [28,29,30,31,32,33,34,35,36,37,38] satisfied all criteria for inclusion. No unpublished studies of relevance were found in ongoing trials, or grey literature searches. Inter-reviewer agreement for inclusion of articles was quite high (*k* = 0.873). Figure 1 depicts the PRISMA flowchart showcasing the literature selection process, adopted to the recommendations of Vu-Ngoc et al. [39].

### 3.2. Study Characteristics

The included studies were all published between 2011 and 2019. While no restrictions had been placed on the search, these parameters were placed to coincide with the last major systematic review published in this field [1]. The previous review included studies up until 2011, so to ensure that no studies in this year were missed, this review incorporates all studies published in 2011. Of the included 11 studies, six reported usage of non-surgical techniques [28,29,30,31,32,33] and five reported on surgical techniques [34,35,36,37,38].

#### 3.2.1. Non-Surgical Techniques

Investigated non-surgical treatment modalities included have been listed in Table 4.

Adjunctive treatments to mechanical debridement were investigated in three studies, including the use of chloramine gel [31], diode laser application [28] and chlorhexidine chips [30].

After a 3-month follow up, Roos-Jansaker et al. found no statistically significant difference between conventional manual debridement (usage of ultrasonic and hand-scalers) and the adjunctive use of chloramine gel in the treatment of peri-implantitis across any of the studied parameters. Despite such an indifference between the groups, both groups resulted in significantly improved clinical outcomes [31]. Similarly, Arisan et al. found no statistically significant differences between conventional debridement and the adjunctive use of a diode laser across many of the studied parameters, including, mean and deepest PPD, plaque index (PI) and BoP. Both modalities showed a statistically significant difference across these parameters from baseline to the study’s conclusion at six months. Marginal bone loss (MBL) was the one parameter where a significant difference was found between the groups, with participants in the laser group revealing greater MBL at the end of the six months as compared to the control, despite no baseline differences [28].

Machtei et al. evaluated the effectiveness of a proposed treatment protocol for peri-implantitis lesions, whereby participants were subjected to intensive repeated applications of chlorhexidine-containing chips in affected sites, as an adjunctive measure to conventional debridement [30]. Patients were randomised to receive either hydrolysed gelatine matrix chips (MatrixC) or a biodegradable matrix containing chlorhexidine chips (PerioC^®®^; Dexcel Pharma, Or-Akiva, Israel). Clinical measurements and chips placements were repeated at weeks 2, 4, 6, 8, 12 and 18 post-intervention, with the participants then returning after 6 months for a final assessment. The study reported that the PPD improvement in the PerioC group was greater than that of the MatrixC group, being borderline significant (*p* = 0.07, mixed model). Clinical attachment level (CAL) gain was also found to be significantly greater in the PerioC group, as compared to the MatrixC group. No in-between group differences were found when measuring BoP, and both groups resulted in significant improvements across all parameters over the 6-month follow up period. Hence, the authors concluded that frequent placement of PerioC and MatrixC, alongside conventional debridement, resulted in substantial improvement in sites affected by peri-implantitis [30].

Three studies investigated the use of air-abrasive devices as a treatment modality, with two comparing them to conventional debridement alongside local chlorhexidine application [28,30], and the other comparing them to an Er:YAG laser application [31].

John et al. found that air-abrasive devices resulted in a significantly higher decrease in mean BoP scores as compared to conventional debridement. However, this result was an anomaly as there were no differences reported across PI, PPD, MR or CAL gains between groups. The study concluded that both treatment options resulted in comparable and significant CAL gains at the end of the 12-month study period, with air-abrasive devices resulting in a greater reduction in BoP [29]. Sahm et al. investigated identical interventions and reached comparable conclusion with their study [32]. They conducted clinical measurements at 3-month and 6-month follow-ups and found that the air-abrasive device group had significantly lower BoP than the conventional debridement group in both time groups, however, there were no other differences across parameters like PI, MR, PPD or CAL gains [32]. Renvert et al. investigated the efficacy of an air-abrasive device as compared to an Er:YAG laser. They found that both therapies allowed statistically significant improvements across all measured parameters, including, BoP, SoP, PI, PPD and MBL. However, they also concluded that there were no inter-group differences between the two treatments across any of the parameters [33].

#### 3.2.2. Surgical Techniques

Investigated surgical treatment modalities have been listed in Table 5.

A combination of resective surgery, an apically positioned flap, bone recontouring and use of either saline [34], placebo [37] or 0.12% CHX and 0.05% CPC solution [38] was investigated in three separate studies. These studies compared this treatment to the additional usage of 35% phosphoric acid [34], combination of 0.12% CHX and 0.05% CPC [37], as well as a stronger 2% CHX solution, respectively [38].

Hentenaar et al. found that adjunctive application of 35% phosphoric acid led to greater immediate reduction in total anaerobic bacterial counts on the implant surface, as well as a significantly lower count of culture-positive DI [34]. Despite showing effective decontamination of the implant surface, phosphoric acid did not present with significantly better clinical or microbiological measurements after the 3-month follow up period, as compared to the control [34]. Similarly, de Waal et al. reported that the adjunctive use of 0.12% CHX + 0.05% CPC resulted in a significantly greater reduction in bacterial load on the implant surface, but did not translate into better clinical results across a 12-month follow up period [36]. The control in this study was a placebo solution [37], and in 2015, de Waal et al. conducted another study, which used the 0.12% CHX + 0.05% CPC as a control to a stronger 2% CHX solution [38]. This study found no significant difference for either microbiological or clinical measurements between the two groups over a 12-month follow-up period. Hence, the authors concluded that a 0.12% CHX + 0.05% CPC successfully reduces anaerobic bacterial load on the implant surface better than debridement alone, but this does not translate to better clinical therapeutic outcomes [38].

Papadopoulos et al. conducted a randomised controlled trial (RCT) investigating the efficacy of using a diode laser as adjunctive treatment to surgical debridement [36]. They performed measurements at baseline, 3 months and 6 months post-intervention. PPD reduction, BoP changes and PI reduction were significant across both groups within the study but did not vary between groups. However, CAL gain was found to be significantly better in the laser group, as compared to the control. The authors concluded that both therapies were equally effective in reducing PPD, BoP and PI, whereas CAL improvement was more associated with the adjunctive use of a diode laser [36].

Isehed et al. investigated the usage of regenerative surgical treatment to treat peri-implantitis lesions, with or without adjunctive enamel matrix derivative (EMD), over a five-year follow-up period [35]. There were no significant differences between the groups at baseline across any of the measured parameters, including, BoP, SoP and MBL. At the conclusion of the study, there were no significant differences between the test group or the control group across any of the aforementioned parameters. At the end of the 5-year period, four of 13 (31%) DI were either lost or retreated due to infection in the EMD group, compared to seven of 12 (58%) in the non-EMD group. Univariate comparisons listed this as an insignificant difference (*p* = 0.48), so the authors ran a partial least square modelling, as this was deemed to be better for smaller samples. Through this model, they were able to conclude that EMD treatment was positively associated with implant survival up to 5 years, but larger studies are required in this field [35].

Table 6 and Table 7 provide a summary of the characteristics of included studies, including each study’s diagnosis of peri-implantitis, number of DI included, interventions, follow-up period, clinical parameters investigated and a summary of the main results.

### 3.3. Data Analysis

Due to the methodological and clinical heterogeneity across included studies, data pooling was not possible. Hence, a narrative synthesis rather than a meta-analysis was conducted.

### 3.4. Risk of Bias

Of the 11 included studies, seven had a low risk of bias in domain one [29,30,33,34,35,37,38], relating to the randomisation process, as opposed to four which presented with some concerns [28,31,32,36], usually due to non-disclosure of critical information.

Regarding the second domain, dealing with deviation from intended intervention, four studies were adjudged to have a high risk of bias [29,31,35,36], two with some concerns [32,34] and five with a low risk [28,30,33,37,38]. High risk was generally associated with a lack of appropriate analysis to deal with missing data and a likely potential of failure to analyse missing data having a significant impact. If the potential of failure to analyse missing data was unlikely to have a significant effect on the studies’ results, then the study was classified as having ‘some concerns.’

Domain three deals with the risk of bias due to missing outcome data. Out of the 11 studies, nine were adjudged to have a low risk of bias [28,29,30,31,32,33,34,37,38], whereas two presented with high risk [35,36]. These studies had a large percentage of participants lost and it was seen to be likely that missingness in the outcome depended on its true value. The fourth domain investigated the risk of bias in measurement of the outcome. Nine of the 11 studies had a low risk of bias in this domain [28,29,30,32,33,34,36,37,38], whereas one caused some concerns [31], and one had high risk [35]. Some concerns were brought about due to the possibility of the outcome being influenced by the knowledge of intervention received, and there was no information reported on whether outcome assessors were aware of the intervention received. However, in the study conducted by Roos-Jansaker et al. [31], it was unlikely that this would have been the case, hence, the study was classified as having some concerns. On the other hand, study by Isehed et al. had different examiners performing clinical measurements, hence measurements could have varied, which resulted in them being adjudged as having a high risk of bias [35].

Finally, the fifth domain referred to the risk of bias in selection of the reported result. Only one study was adjudged as having some concerns in this regard [28], whereas all the others had low risk [29,30,31,32,33,34,35,36,37,38]. In Arisan’s study, the method specifies the usage of ANOVA for the MBL and PPD values, however, the results only show the MBL values and not the PPD values [28].

Overall, four studies had a low risk of bias [30,33,37,38], three had some concerns [28,32,34] and four had a high risk [29,31,35,36]. These results are depicted in Table 8.

### 3.5. Quality of the Evidence

The GRADE tool was utilised to ascertain the overall quality of evidence provided, through the assessment of criteria including risk of bias, inconsistency, indirectness, imprecision and publication bias. Four interventions were adjudged to have presented a low quality of evidence, whereas the other nine were all very low quality. Due to this, the true effect of an intervention is likely to be substantially removed from the estimated effect for most interventions. These results, alongside reasons for these judgements, are denoted in Table 9. There was a high agreement amongst reviewers regarding the GRADE assessment, reflected in a kappa value of 0.839.

## 4. Discussion

In 2018, Ting et al. conducted a systematic review of peri-implantitis-related systematic reviews and meta-analyses, in order to provide a comprehensive overview of peri-implantitis [40]. This review concluded that “no strong evidence was found to suggest the most effective treatment intervention for peri-implantitis, although most peri-implantitis therapies can produce successful outcomes” [40].

In recent decades, it has become critical that clinicians limit their prescription of systemic antibiotics to deter the development of antibiotic resistance and super-infections [22,41]. Adjunctive systemic antibiotic usage has been suggested as a therapeutic measure for peri-implantitis lesions, however, their benefit is questionable [42,43]. Despite their usage, retreatment is often required, indicating that their usage should be restricted [44,45]. In light of these findings, such a systematic review was conducted to allow clinicians to appreciate the most effective debridement and/or decontamination techniques in treating peri-implantitis lesions.

### 4.1. Overall Completeness and Applicability of Evidence

Of the eleven included studies, four were deemed to have a high risk of bias [29,31,35,36] and three presented with certain concerns [28,32,34]. The completeness of all studies will be discussed across the following parameters in order to help clinicians better evaluate these RCTs:

#### 4.1.1. Inclusion Criteria

In their systematic review encompassing studies from 1950 to 2011, Esposito et al. reported that some of their included studies did not adequately describe the initial degree of pathology around included DI, particularly regarding MBL [1]. Some studies had to be as they failed to distinguish between peri-implant mucositis and peri-implantitis, thus confounding the results. However, this was not an issue in this review, as all studies had clearly defined their diagnostic determinants of peri-implantitis, and all included some form of bone loss. This could possibly be due to the greater knowledge available surrounding this topic in later years [2,3].

#### 4.1.2. Exclusion Criteria

To be a part of this review, any studies in which patients had been prescribed antibiotics pre-operatively, peri-operatively or post-operatively were excluded. Apart from this, common exclusions included: patients with a history of periodontitis, systemic diseases which can affect peri-implantitis treatment (uncontrolled diabetes, osteoporosis, cardiovascular diseases etc.) [28,29,30,31,32,33,34,35,36,37,38]; some studies also excluded smokers [27,30]. While Esposito et al. suggested that such strict exclusion criteria may limit the extrapolation of the findings to a broader population [1], it does help in reducing confounders to the treatment and evaluating the efficacy of the studied interventions.

#### 4.1.3. Outcome Measures

While the main outcome measure for successful treatment of peri-implantitis lesions could be implant retention, it was only specifically reported as an outcome measurement in one study [35]. On the other hand, MBL is regarded as the most reliable prognostic tool for treatment, but it was only used as an outcome measurement in one non-surgical study [26] and three surgical studies [35,37,38]. These results are strikingly similar to the findings of Esposito et al. [1] suggesting that the exploration of implant failure and MBL could be better used as outcome measurements in trials of this nature.

#### 4.1.4. Interventions

Several interventions were investigated in the included studies. Most had been suggested from previous clinical experience trying to parallel treatment of peri-implantitis with the treatment of periodontitis. In this case, all included trials involved appropriate interventions and most studies provided a justification for their choice of intervention. It has been suggested that extensive treatment may confound treatment outcomes, making it difficult to distinguish between effective and non-effective components of treatment [1]. Some studies had funding from the manufacturers of the interventions that they employed [29,31,32,33], while others had instruments supplied from the manufacturer [38] or had been part of the manufacturer’s research and development team [30]. However, none of the studies explicitly declared a conflict of interest [1].

#### 4.1.5. Sample Sizes

Sample sizes across most studies included in this review were small, with the largest study managing to recruit 60 patients [30] and the smallest study recruiting 18 patients [31]. With an increasing proportion of individuals being rehabilitated with DI, and DI being in function for an increased number of years, the number of patients who are affected by peri-implantitis will inevitably increase in absolute terms. Hence, future studies must aim to recruit higher numbers of individuals and eligible implant sites to provide higher levels of evidence with minimal risk of bias.

#### 4.1.6. Randomisation and Blinding

To ensure highest level of evidence is being investigated, it is critical that authors of RCTs clearly outline their randomisation techniques and ensure blinding of participants and clinicians to the highest possible degree. As four studies in this review failed to reveal the exact nature of their blinding [28,31,32,36], they were adjudged to have some concerns regarding their risk of bias. More strenuous effort is required to ensure that appropriate study designs are being implemented.

#### 4.1.7. Follow-Up

Peri-implantitis is a chronic disease, with an increased risk for those DI which have been in function for greater than five years. Its treatment is also long-term and needs to be followed-up for a long period of time. Most studies included in this review only had follow-up periods of 6–12 months, which is a relatively short follow-up time for such a chronic disease; two trials had a follow-up of just 3 months [31,34]. Only one study had a follow-up period of longer than 12 months, which was 5 years [35]. Studies with higher follow-up periods are vital in evaluating the long-term effects of treatment modalities.

#### 4.1.8. Statistical Analysis

Most studies included in this review included DI as the unit of statistical analysis, rather than the number of patients. This poses an issue, as patient factors play a critical role towards successful treatment. Treatment outcomes of multiple implicated DI in a single patient are dependent on multiple factors, including patient’s oral hygiene, design of the prosthesis, systemic health and smoking status [1]. It has been suggested that trials adopt a split-mouth or parallel group design or include the clustering of DI within a patient during the analysis. Multilevel modelling has been suggested as a method to carry out such a statistical analysis [1]. Most studies included in this review performed some form of multilevel modelling, being able to adjust for confounders and trying to find associations, which is an improvement from previous reviews.

#### 4.1.9. Drop Outs, Withdrawals and Failures

Patients being lost to follow-up is a common occurrence in clinical studies. However, there is a requirement that any patient lost due to any reason, should be accounted for and reported in the final report. In this case, if treatment fails, and further treatment is required, or an explantation is required, this must be reported and appropriately accounted for in the statistical analysis. Most studies in this review often excluded any individuals who had failing DI from their final calculations and did not have appropriate statistical methodology to account for the missing data. Due to this reason, several studies were found to have a high risk of bias [29,31,35,36], or at least some concerns [32,34], downgrading their reliability.

### 4.2. Quality of the Evidence

While this review has been conducted to ensure that the highest-level of evidence is included and assessed, it has become apparent that there is a lack of high-quality clinical trials in this area. All interventions have been assessed as being of low or very low quality of evidence, suggesting that the true effect of each intervention may be substantially different from the estimated effect. Of the evaluated interventions, only four interventions were deemed to be of low quality, rather than very low quality. These interventions included:

1. Non-surgical debridement + CHX chips;
2. Non-surgical debridement + matrix chips;
3. Surgical resective treatment + 0.12% CHX and 0.05% CPC;
4. Surgical resective treatment + 2% CHX.

These interventions came from three out of the eleven investigated studies [30,37,38], showing a poor quality of evidence compared to eight other studies [28,29,31,32,33,34,35,36]. These low-quality interventions, like all the others, also suffered from a downgrade due to low sample sizes and publication bias. In this GRADE analysis, number of DI has been utilised as a unit, but if patients were used, the sample sizes would be even lower. The need for higher sample sizes to ensure that high-level evidence has been employed is vital if a true gold standard therapy for peri-implantitis debridement and/or decontamination is to be ascertained.

### 4.3. Practical Implications

#### 4.3.1. Non-Surgical Therapies

The adjunctive use of chloramine gel as a decontaminant was equally as effective as non-surgical debridement therapy alone, resulting in statistically significant clinical improvements for a follow-up period of 3 months [31]. The study recruited 18 individuals with at least two DI impacted by peri-implantitis and exposed one to the control and one to the test. This low sample size, combined with a short follow-up period of 3 months, and the fact that an appropriate analysis was not used to estimate the effect of assignment led to the study being adjudged to have a high risk of bias, as well as very low-quality evidence for both interventions. There is scope for further research in this field, preferably an independent study, with a larger number of participants, clearer methodology and longer follow-up period.

Machtei et al. investigated the efficacy of repeated applications of matrix (MatrixC) and chlorhexidine-containing chips (PerioC) following non-surgical treatment [30]. They reported that the PerioC led to a mean PPD reduction of 2.19 mm, as compared to 1.59 mm in MatrixC, and a CAL gain of 2.21 mm as compared to 1.56 mm in the MatrixC group. Both parameters were significantly better in the PerioC group, however, there were no group differences in the BoP over the 6-month follow-up period. The authors reported that this was a better than expected result [30], as a meta-analysis of non-surgical treatment had concluded that a mean PPD reduction of 0.77 mm and CAL gain of 0.79 mm can be expected from non-surgical therapy [46]. Machtei et al. also predicted that the significant improvement in the MatrixC group suggested that the matrix degradation itself had an antibacterial effect [30]. This study was adjudged to have a low risk of bias, and hence the interventions presented were rated as low-quality evidence, being penalised for small sample sizes. Despite this, the study was well conducted and presented with positive results, which will require further evaluation.

Arisan et al. found that the adjunctive use of a diode laser was equally as effective across most clinical parameters, as compared to the control, but was found to have led to higher MBL than the control group, despite there being no difference at baseline [28]. The authors suggest that this could be due to several factors, including, individual host response, confounding factors in the healing mechanism of the peri-implant alveolar bone, or even a negative impact of the diode laser. It has been suggested that excessive thermal damage due to the laser application might jeopardize healing conditions [47,48]. The authors had tried to be mindful of this, by utilising a low-powered 810 nm diode laser, which has previously been shown to be innocuous on the implant surface [49], on top of utilising similar methodology to previous studies, but cannot rule out this possibility. Unfortunately, their small sample size and concerning risk of bias assessment means that both interventions were assessed to be of very low quality [28], hence, their results cannot be used for a generalised conclusion.

Prior to study conducted by Arisan et al., a systematic review investigated the effect of various laser wavelengths in the treatment of peri-implantitis [50]. They reported that non-surgical laser treatment with a single application of either the Er:YAG (2940 nm) laser or a diode (660 nm) laser in combination with a phenothiazine chloride dye was efficient in controlling inflammation around treated DI for at least 6 months following intervention. However, this treatment had limited effects on reduction in PPD and gain in CAL. Based on this, the authors cautiously concluded that laser treatment may be further investigated as phase 1 therapy for the treatment of peri-implantitis, but based on their findings, no superiority of laser treatment above conventional treatment could be found.

Renvert et al. investigated the efficacy of an Er:YAG laser against an air-abrasive device [33]. They found that both treatment outcomes were equally effective in reducing peri-implantitis across the measured parameters, but no statistically significant differences between the groups were evident. While there were little group differences, they found that the overall clinical improvement across both modalities was limited, and hence, concluded that both treatment modalities are insufficient to treat deep peri-implantitis defects. The authors suggested that this may be due to the severity of the disease and further studies would need to be conducted to determine whether there are threshold levels beyond which non-surgical intervention is ineffective. They also acknowledged that another possibility could include tissue trauma for the treatment, but this was unlikely as they used laser settings below defined risk values [51], as well as a protocol for appropriate usage of the air-abrasive device in periodontal pockets [52], and no serious adverse events were recorded across either group. The authors acknowledged that the lack of eligible participants is a chronic issue in clinical dentistry research, but otherwise, this study was assigned a low risk of bias. However, due to the methodology of other studies investigating the same interventions, both interventions’ overall GRADE were of a very low quality.

Both studies by Sahm et al. and John et al. investigated the efficacy of air-abrasive devices against the control of manual debridement and local application of chlorhexidine. Both studies reached the same conclusion, with both treatment modalities being similar in their efficacy across PPD reductions and CAL gains, but the air-abrasive device leading to a statistically greater suppression of BoP. This parallels the conclusion drawn by the systematic review conducted by Shwarz et al. regarding the efficacy of air polishing for the non-surgical treatment of peri-implant diseases [22]. Sahm et al. put this effect down to the increased effectiveness of air-abrasive devices over manual debridement in removing bacterial plaque biofilms [53,54,55], hence, reducing bacterial load and leading to lower BoP scores.

The use of non-surgical manual debridement with chlorhexidine disinfection revealed limited clinical efficacy in controlling disease progression, in line with other studies of the same nature [56,57]. Sahm et al. [32] differentiated their results from Renvert et al. [33] who reported that air-abrasive devices fared no better than Er:YAG laser treatment, by pointing out that Sahm et al. included patients suffering from initial to moderate peri-implantitis, rather than severe peri-implantitis. This suggests that the efficacy of non-surgical treatment with adjunctive air-abrasive devices may be limited to a threshold level. John et al. proved that these results could be maintained for 12 months, although both studies suffered from concerns and a high risk of bias, respectively. All these studies were industry funded. This fact, alongside the high risk of bias and small sample sizes, resulted in the intervention being graded as very low-quality.

#### 4.3.2. Surgical Therapies

Hentenaar et al. compared the adjunctive 35% phosphoric acid to resective therapy and decontamination with saline solution [34]. While they found that the test group led to a significantly greater immediate suppression of anaerobic bacterial counts on the implant surface, this did not translate into better clinical results or even microbiological results by the end of the three-month follow up period. The authors chose phosphoric acid as a decontaminating agent as acids with low pH have been reported to exert a potent bactericidal effects [56,57], and have an innocuous effect on the titanium implant surface [58]. While their study found that the adjunctive usage of 35% phosphoric acid had no net positive or negative effect on top of surgical resective treatment, the authors hypothesised that surface damage of dental alloys may potentially be induced after detoxification with acidic solutions, hindering re-osseointegration. Wheelis et al. reported noticeable morphological changes and corrosion on the titanium surface when the synergistic effect of acidic environments and mechanical forces were investigated, as opposed to neutral and basic treatments [59]. There were concerns regarding risk of bias in this study, in combination with a small sample size and a short follow-up period of 3 months, accumulating in both interventions ultimately being graded as being of very low quality.

Similarly, de Waal et al. discovered that a 0.12% CHX + 0.05% CPC solution lead to greater reduction in bacterial load on the implant surface, but this did not translate into a better clinical or radiographical results over the 12-month follow-up [37]. They compared this to the results of Schwarz et al. who also found that the method of surface debridement and decontamination did not impact the clinical outcomes following combined surgical therapy of advanced peri-implantitis lesions [60,61]. The authors suggested that the long-term stability of clinical outcomes may be influenced by factors other than the method of decontamination. The authors chose to use a combination of 0.12% CHX and 0.05% CPC non-alcoholic solution in this study as it has been reported to be an equally effective anti-plaque and anti-inflammatory agent as the 0.2% CHX solution with alcohol [62]. In 2015, de Waal et al. extended their research by comparing the efficacy of 2% CHX solution against 0.12% CHX + 0.05% CPC adjunctive to resective surgical treatment [38]. They found no significant differences between either group over three follow-up periods (3, 6 and 12 months) over microbiological or clinical parameters, despite both being effective. The authors concluded that despite the 2% CHX solution not causing any detrimental clinical effects, it neither contributed to improved clinical or radiographic outcomes, despite being over 16 times the strength of the control solution, suggesting a threshold level for efficacy. Both studies were assessed to have a low risk of bias and were adjudged to be of low quality only due to the small sample sizes for each intervention.

Papadopoulos et al. investigated the adjunctive use of a diode laser to surgical treatment and discovered that the diode laser results in a significantly greater CAL, but no other significant advantage over surgical treatment [36]. Both treatments were equally effective across all other parameters and hence the authors concluded that the additional use of a diode laser offers limited clinical benefit. The authors suggested several confounding variables which could contribute to a variety of non-comparable results across studies of similar nature, including type of lasers, frequency of laser irradiation, different kinds of peri-implantitis lesions, removal of supra-structures and application of implantoplasty. Despite this result, this study’s high risk of bias, combined with small sample size resulted in both interventions being assessed as being of very low quality.

Isehed et al. investigated the efficacy of surgical regenerative treatment over an extended follow-up of 5-years [35]. They concluded that adjunctive enamel matrix derivative (EMD) usage was positively associated with implant survival. The authors acknowledged that the removal of DI with the most advanced bone loss probably influenced the results, despite there being a significant increase of approximately 1 mm of bone after 5-years across both groups. They also discovered that some changes, seen at the 1-year follow-up, disappeared at the consequent 3-year and 5-year follow-ups, suggesting a single treatment may not be sufficient to ensure a stable long-term result [63,64]. In the EMD group, 11/13 DI (84.6%) survived compared to 9/12 in the non-EMD group (75%). The authors predicted that this could be due to EMD-induced early healing and an additional anti-microbial effect [65,66]. Despite a long-term follow-up unlike most other studies included in this review, this study suffered from a high risk of bias, small sample size and hence was adjudged to present very low-quality evidence.

## 5. Conclusions

As an increasing proportion of the global population gets edentulous spaces rehabilitated by DI, the absolute incidence of peri-implantitis is expected to rise. With an ever-increasing global threat of super-infections and antibiotic resistance, it is essential that treatment which minimises harmful excessive usage of antibiotics is adopted. Hence, the need to study the efficacy of techniques used to manage peri-implantitis lesions without antibiotics is critical.

Despite this, the standard of research that is being presented in this field is currently lacking in the quality needed to make assertive conclusions about the efficacy of any treatment. With many studies lacking appropriate methodological quality, lengthy follow-up periods and large sample sizes, it is difficult to rely upon the results of the studies that have been published in this field.

The current review suggests that a surgical approach to treating peri-implantitis lesions in the absence of antimicrobial therapy may have greater efficacy than a non-surgical approach. Despite this weak indication, the authors of this review recommend further investigation of both approaches and adjunctive measures to determine the best treatment protocol for managing peri-implantitis lesions.

## Figures and Tables

**Table 1:** Treatment for peri-implantitis [11].

Treatment for Peri-Implantitis
Non-surgical therapy to remove local irritants from the implant’s surface with or without: Surface decontamination Additional adjunctive therapies Surgical therapy to remove any residual subgingival deposits and reduce peri-implant pocket depths with or without: Resective Osseous therapy Regenerative Osseous therapy Additional adjunctive therapies [11]

**Table 2:** Eligibility Criteria.

	Inclusion Criteria	Exclusion Criteria
Population	Healthy human patients receiving treatment for peri-implantitis lesions	Studies of human patients with chronic diseases, co-morbidities and non-human studies; Animal studies
Intervention	Surgical or non-surgical treatment of peri-implantitis lesions, including adjunctive treatment	Studies which allow/use pre-operative (up to 3 months prior to initiation), peri-operative and post-operative anti-microbial therapy
Comparator	Individuals or teeth within the same individual (including split mouth technique) not subjected to the same therapeutic variable	
Outcome	Resolution of peri-implantitis, including implant survival and absence of peri-implant probing pocket depths of >5 mm, suppuration, bleeding on probing (BoP) and further bone loss	Studies including patients who have previously received peri-implantitis treatment
Study Design	Randomised controlled trials (RCT), published or unpublished	Non-RCT, cohort studies, case reports, case series, reviews, abstracts, systematic reviews, opinions, studies with questionnaires or studies where the diagnosis/measurement of peri-implantitis was performed only on radiographs rather than clinically.

**Table 3:** Search Strategy for Medline via OVID.

#	Searches	Results
1	exp Peri-implantitis/	1017
2	exp Therapeutics/	4,367,916
3	exp Operative Surgical Procedures/	2,990,228
4	2 OR 3	6,056,211
5	1 AND 4	453

**Table 4:** Non-surgical treatment modalities.

Mechanical debridement versus adjunctive diode laser application [28] Mechanical debridement + topical chlorhexidine (CHX) application versus air-abrasive device containing amino acid glycine powder [29,32] Mechanical debridement + matrix chips vs. mechanical debridement + CHX chips [30] Er: YAG laser versus air-abrasive device containing hydrophobic powder [33] Mechanical debridement versus adjunctive local applications of chloramine gel [31]

**Table 5:** Surgical treatment modalities.

Resective surgery + apically positioned flap + bone recontouring + sterile saline versus resective surgery + apically positioned flap + bone recontouring + 35% phosphoric acid [34] Regenerative surgical treatment versus regenerative surgical treatment + enamel matrix derivative [35] Access flap + plastic curette debridement + saline versus access flap + plastic curette debridement + diode laser [36] Resective surgery + apically positioned flap + bone recontouring + debridement + placebo versus resective surgery + apically positioned flap + bone recontouring + debridement + 0.12% CHX + 0.05% cetylpyrinidium chloride (CPC) [37] Resective surgery + apically positioned flap + bone recontouring + debridement + 0.12% CHX versus resective surgery + apically positioned flap + bone recontouring + debridement + 2% CHX [38]

**Table 6:** Summary of non-surgical studies.

References	Diagnosis of Peri-Implantitis	No. of Implants	Treatment Strategies		Follow-Up	Study Parameters	Results
			Group 1	Group 2			
Arisan et al. (2015)	4–6 mm of PPD <3 mm of MBL BoP, Plaque, Pain and/or SoP	48	Diode Laser	Mechanical debridement	6 months	PPD PI BoP MBL Bacterial Count	Adjunctive use of a diode laser did not yield any additional positive influence on the peri-implant health compared with conventional scaling alone
John et al. (2015)	PPD ≥ 4 mm BoP SoP MBL ≤ 30%	25	Amino Acid Glycine Powder (AAD)	Mechanical Debridement with carbon curettes + Antiseptic therapy chlorhexidine (MDA)	12 months	PI BoP PPD Mucosal Recession CAL	Both treatment procedures resulted in comparable but limited CAL gains; AAD was assoc. significantly higher BoP than MDA
Machtei et al. (2012)	PPD of 6–10 mm BoP MBL	73	Matrix Chips (MatrixC)	Chlorhexidine Chips (PerioC)	6 months	PPD change CAL BoP	Both groups resulted in substantial improvement; CAL changes in PerioC group were significantly greater than MatrixC
Renvert et al. (2011)	PPD ≥ 5 mm Bone Loss ≥ 2 mm BoP	100	Er:YAG Laser	Air-Abrasive device	6 months	PPD BoP Bacterial Counts	Both methods showed limited clinical improvement, but failed to reduce bacterial count
Roos-Jansaker et al. (2017)	MBL ≥ 2 mm PPD > 4 mm BoP and/or SoP	32	Local applications of chloramine gel Supra- and submucosal debridement by ultrasonic and hand instruments	Supra- and submucosal debridement by ultrasonic and hand instruments	3 months	PI PPD CAL BoP	Adjunctive use of chloramine is equally effective in the reduction in mucosal inflammation as conventional non-surgical mechanical debridement up to 3 months
Sahm et al. (2011)	PPD ≥ 4 mm Bone Loss ≤ 30% BoP SoP No occlusal overload 2 mm keratinized mucosa Good PI	43	Amino Acid Glycine Powder (AAD)	Mechanical Debridement with carbon curettes + Antiseptic therapy chlorhexidine (MDA)	6 months	BoP PPD CAL	Both groups revealed comparable PD reduction and CAL gains Higher changes in BoP in the AAD group

PPD—peri-implant probing depth; BoP—Bleeding on Probing; SoP—Suppuration on Probing; MBL—Marginal Bone Loss; CAL—Clinical Attachment Loss; PI—Plaque Index.

**Table 7:** Summary of surgical studies.

References	Diagnosis of Peri-Implantitis	No. of Implants	Treatment Strategies		Follow-Up	Study Parameters	Results
			Group 1	Group 2			
Hentenaar et al. (2017)	MBL ≥ 2 mm PPD ≥ 5 mm BoP and/or SoP	50	Resective surgery with apically positioned flap Bone recontouring 35% phosphoric acid etching gel	Resective surgery with apically positioned flap Bone recontouring Sterile Saline	3 months	Bacterial count BoP SoP Mean PPD	35% phosphoric acid led to greater decontamination of the implant surface, but did not enhance clinical outcomes
Isehed et al. (2018)	PPD ≥ 5 mm BoP and/or SoP Angular bone loss ≥ 3 mm	14	Regenerative surgical treatment with adjunctive enamel matrix derivative (EMD)	Regenerative surgical treatment	5 years	Implant loss MBL BoP Plaque SoP	Adjunctive EMD is positively associated with implant survival up to 5 years
Papadopoulos et al. (2015)	PPD ≥ 6 mm BoP SoP MBL ≥ 2 mm	16	Access flap Plastic curette Sterilised gauze soaked in saline	Access flap Plastic curette Diode Laser	6 months	PPD CAL BoP PI	Surgical treatment leads to improvement of all clinical parameters; additional use of diode laser does not have beneficiary effect
de Waal et al. (2013)	PPD ≥ 5 mm Bone loss ≥ 2 mm BoP and/or SoP	79	Resective surgery with apically positioned flap Bone recontouring Debridement 0.12% CHX 0.05% CPC	Resective surgery with apically positioned flap Bone recontouring Debridement Placebo	12 months	Bacterial Count PI BoP SoP PPD MBL	CHX + CPC leads to greater immediate suppression of bacterial load, but this does not translate into better clinical results
de Waal et al. (2015)	PPD ≥ 5 mm Bone loss ≥ 2 mm BoP and/or SoP	102	Resective surgery with apically positioned flap Bone recontouring Debridement 2% CHX	Resective surgery with apically positioned flap Bone recontouring Debridement 0.12% CHX 0.05% CPC	12 months	BoP PI SoP PPD MBL Bacterial Count	2% CHX does not lead to improved clinical, radiographic or microbiological results compared with a 0.12% CHX and 0.05% CPC solution

PPD—peri-implant probing depth; BoP—Bleeding on Probing; SoP—Suppuration on Probing; MBL—Marginal Bone Loss; CAL—Clinical Attachment Loss; PI—Plaque Index; CHX—Chlorhexidine; CPC—Cetylpyrinidium Chloride.

**Table 8:** Cochrane Risk of Bias Tool.

Study/Domain	Domain 1	Domain 2	Domain 3	Domain 4	Domain 5	Overall
Arisan 2015	Concerns	Low	Low	Low	Concerns	Concerns
Hentenaar 2017	Low	Concerns	Low	Low	Low	Concerns
Isehed 2018	Low	High	High	High	Low	High
John 2015	Low	High	Low	Low	Low	High
Machtei 2012	Low	Low	Low	Low	Low	Low
Papadopoulos 2015	Concerns	High	High	Low	Low	High
Renvert 2011	Low	Low	Low	Low	Low	Low
Roos-Jansaker 2017	Concerns	High	Low	Concerns	Low	High
Sahm 2011	Concerns	Concerns	Low	Low	Low	Concerns
De Waal 2013	Low	Low	Low	Low	Low	Low
De Waal 2015	Low	Low	Low	Low	Low	Low

**Table 9:** GRADE Assessment.

Intervention	No. of Implants (Studies)	Risk of Bias	Inconsistency	Indirectness	Imprecision	Publication Bias	Effect	Overall Certainty of Evidence
Non-surgical manual debridement of implant surfaces	40 (2 Studies)	Very Serious ^1^	Not serious	Not serious	Serious ^3^	Suspected ^4^	All studies report positive changes to clinical parameters surrounding peri-implantitis lesions	⊕○○○ VERY LOW
Surgical debridement of implant surfaces	71 (4 studies)	Very Serious ^1^	Not serious	Not serious	Serious ^3^	Suspected ^4^	All studies report positive changes to clinical parameters surrounding peri-implantitis lesions	⊕○○○ VERY LOW
Non-surgical debridement + Diode laser	45 (2 studies)	Serious ^2^	Not serious	Not serious	Serious ^3^	Suspected ^4^	All studies report positive changes to clinical parameters surrounding peri-implantitis lesions	⊕○○○ VERY LOW
Adjunctive Diode Laser Application with surgical debridement	8 (1 study)	Very Serious ^1^	Not serious	Not serious	Serious ^3^	Suspected ^4^	Single study reports positive changes to clinical parameters surrounding peri-implantitis lesions, however, additional usage of diode laser does not have significant benefit	⊕○○○ VERY LOW
Surgical Debridement + Phosphoric Acid Decontamination	30 (1 study)	Serious ^2^	Not serious	Not serious	Serious ^3^	Suspected ^4^	Single study reports greater decontamination of implant surface with phosphoric acid, however, no clinical benefit seen as compared to control	⊕○○○ VERY LOW
Surgical debridement + Enamel Matrix Derivative (EMD)	9 (1 study)	Very Serious ^1^	Not serious	Not serious	Serious ^3^	Suspected ^4^	Single study suggests that usage of adjunctive EMD is associated with greater implant survival up to 5 years	⊕○○○ VERY LOW
Non-surgical debridement + chlorhexidine chips	40 (1 study)	Not serious	Not serious	Not serious	Serious ^3^	Suspected ^4^	Single study reports that CHX chips result in substantial improvement in sites with peri-implantitis	⊕⊕○○ LOW
Non-surgical debridement + matrix chips	33 (1 study)	Not serious	Not serious	Not serious	Serious ^3^	Suspected ^4^	Single study reports that matrix chips result in substantial improvement in sites with peri-implantitis	⊕⊕○○ LOW
Non-surgical debridement + Air-abrasive device	48 (3 studies)	Very Serious ^1^	Not serious	Not serious	Serious ^3^	Suspected ^4^	All studies report positive changes to clinical parameters surrounding peri-implantitis lesions	⊕○○○ VERY LOW
Non-surgical debridement + chloramine gel	16 (1 study)	Very Serious ^1^	Not serious	Not serious	Serious ^3^	Suspected ^4^	Single study reports improvements in some clinical parameters surrounding peri-implantitis lesions, however, no group difference was found between conventional debridement and adjunctive use of chloramine	⊕○○○ VERY LOW
Non-surgical debridement + chlorhexidine application	28 (2 studies)	Very Serious ^1^	Not serious	Not serious	Serious ^3^	Suspected ^4^	All studies report positive changes to clinical parameters surrounding peri-implantitis lesions	⊕○○○ VERY LOW
Surgical resective treatment + 0.12% CHX and 0.05% CPC	80 (2 studies)	Not serious	Not serious	Not serious	Serious ^3^	Suspected ^4^	All studies report positive changes across several indicators of peri-implantitis	⊕⊕○○ LOW
Surgical resective treatment + 2% CHX	49 (1 study)	Not serious	Not serious	Not serious	Serious ^3^	Suspected ^4^	Single study reported positive changes across several indicators of peri-implantitis, however, no significant difference between solutions of 2% CHX against 0.2% CHX + 0.05% CPC	⊕⊕○○ LOW
The outcome of interest: resolution of peri-implantitis (for which a single pooled effect estimate was not available and only a narrative synthesis of the evidence was provided).								

NOTE: As the outcome for all interventions was the resolution of peri-implantitis lesions, individual GRADE Summary of Findings tables were collated into a single table for publication purposes. GRADE Working Group grades of evidence. High quality: Further research is very unlikely to change our confidence in the estimate of effect. Moderate certainty: Further research is likely to have an important impact on our confidence in the estimate of effect and may change the estimate. Low certainty: Further research is very likely to have an important impact on our confidence in the estimate of effect and is likely to change the estimate. Very low certainty: Any estimate of effect is very uncertain. ^1^ The evidence was downgraded by two levels because of very serious concern regarding the risk of bias; one or more included studies have high risk of bias. ^2^ The evidence was downgraded by one level because of serious concern regarding the risk of bias; one or more included studies have raised some concerns regarding risk of bias. ^3^ The evidence was downgraded by one level because the results came from small studies and numbers of participants, with insufficient event rates for dichotomous and continuous outcomes. ^4^ The evidence was downgraded by one level because of results came from small studies with small numbers of participants.
